# Supplementary figures and images for: Post-infection treatment with the E protein inhibitor BIT225 reduces disease severity and increases survival of K18-hACE2 transgenic mice infected with a lethal dose of SARS-CoV-2
Source: PLoS Pathog. 2023 Aug 7;19(8):e1011328. doi: 10.1371/journal.ppat.1011328 (PMC10434922; doi:10.1371/journal.ppat.1011328)

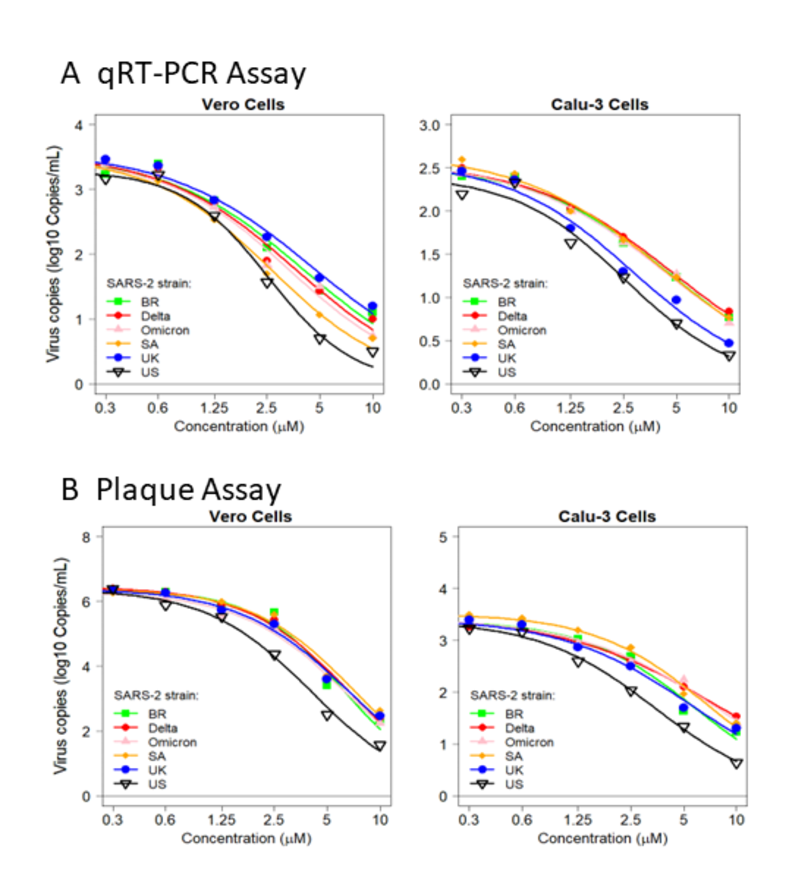

Supplement: S1 Fig — Monolayers of Calu-3 cells (A & B, right panels) or Vero E6 cells (A & B, left panels)–in triplicate wells—were pre-exposed to one of 6 concentrations of BIT225 for 1 hour, then infected (m.o.i = 0.1) with one of 6 SARS-CoV-2 strains: “BR” (green squares—Japan/TY7-503/2021-Brazil_P.1), “Delta” (red circles—US-PHC658/2021), “omicron” (pink triangles—US/MD-HP20874/2021), “SA” (orange diamonds—SouthAfrica/KRISP-K005325/2020), “UK” (blue circles—England/204820464/2020), and “US” (open black triangles—US-WA1/2020). After 4 days, culture media were harvested and assayed for viral load by qRT-PCR (A); or for infectious virus titre by plaque assay (B). Log-logistic dose response curves were fit as described in Methods. EC50, maximum response and Hill slope values are listed in Tables 1 and S1. (TIF) [file ppat.1011328.s001.tif]

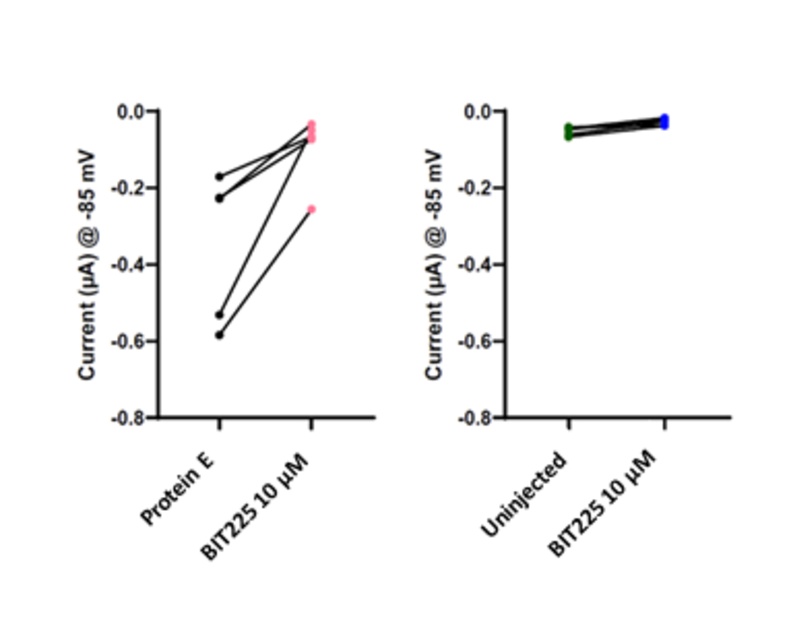

Supplement: S2 Fig — Change in currents for n = 5 individual oocytes after application of BIT225 (10 μM) at -85 mV holding potential: Injected (left panel) and uninjected (right panel). (TIF) [file ppat.1011328.s002.tif]

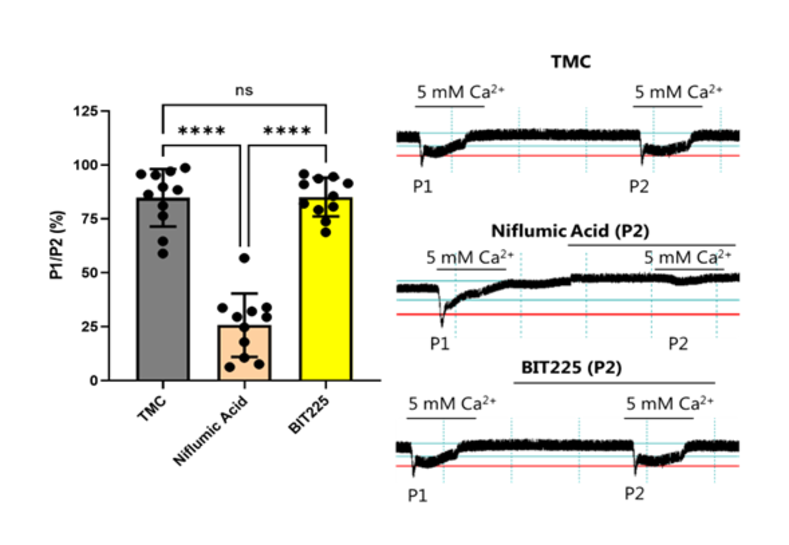

Supplement: S3 Fig — Empty oocytes were exposed to 5 mM Ca2+ solutions in the absence (P1) and presence (P2) of drug and the Ca2+-induced currents were recorded. (A) The effect of drug treatment on the ratio of P1 and P2 currents relative to untreated control. (B) Representative traces of Ca2+-induced currents in the absence (P1) and presence (P2) of the indicated compound. (TIF) [file ppat.1011328.s003.tif]

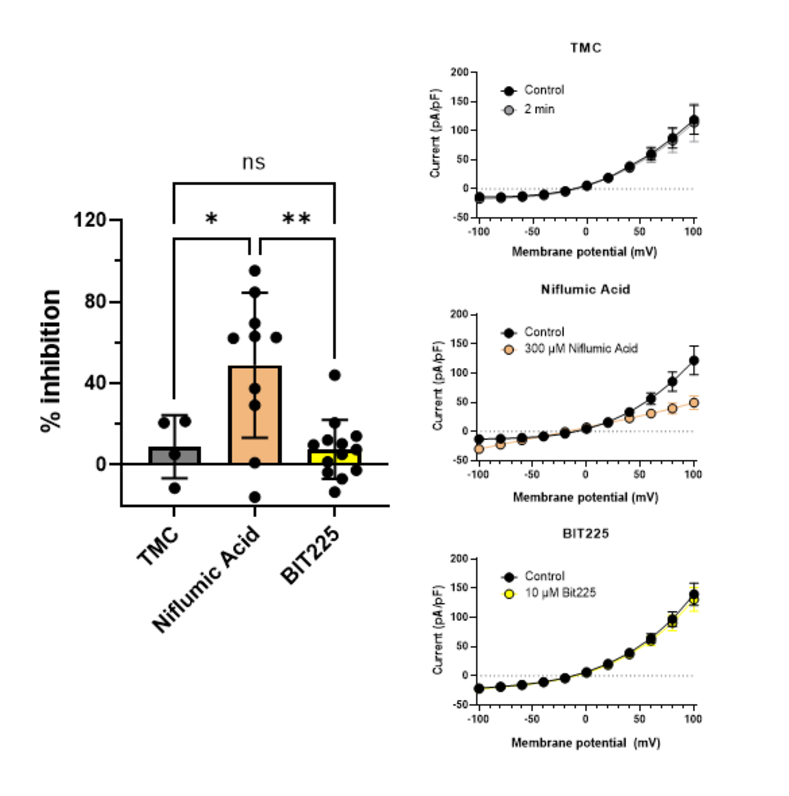

Supplement: S4 Fig — The Xenopus Ca2+-induced ion channel TMEM16A was transiently expressed in HEK293 cells. Ion channel activity was measured by manual patch clamp in the absence and presence of inhibitors as indicated. (TIF) [file ppat.1011328.s004.tif]

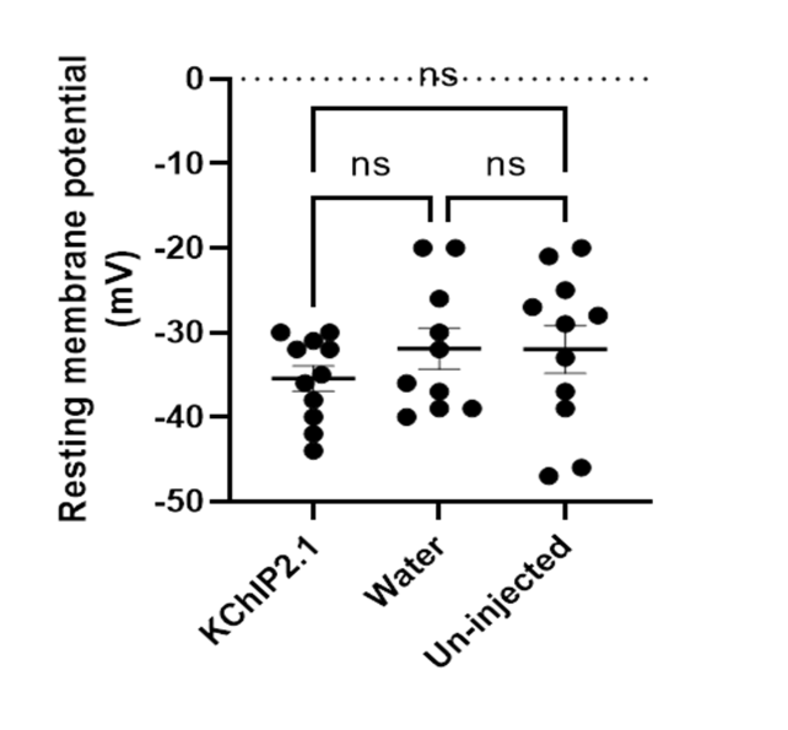

Supplement: S5 Fig — (TIF) [file ppat.1011328.s005.tif]

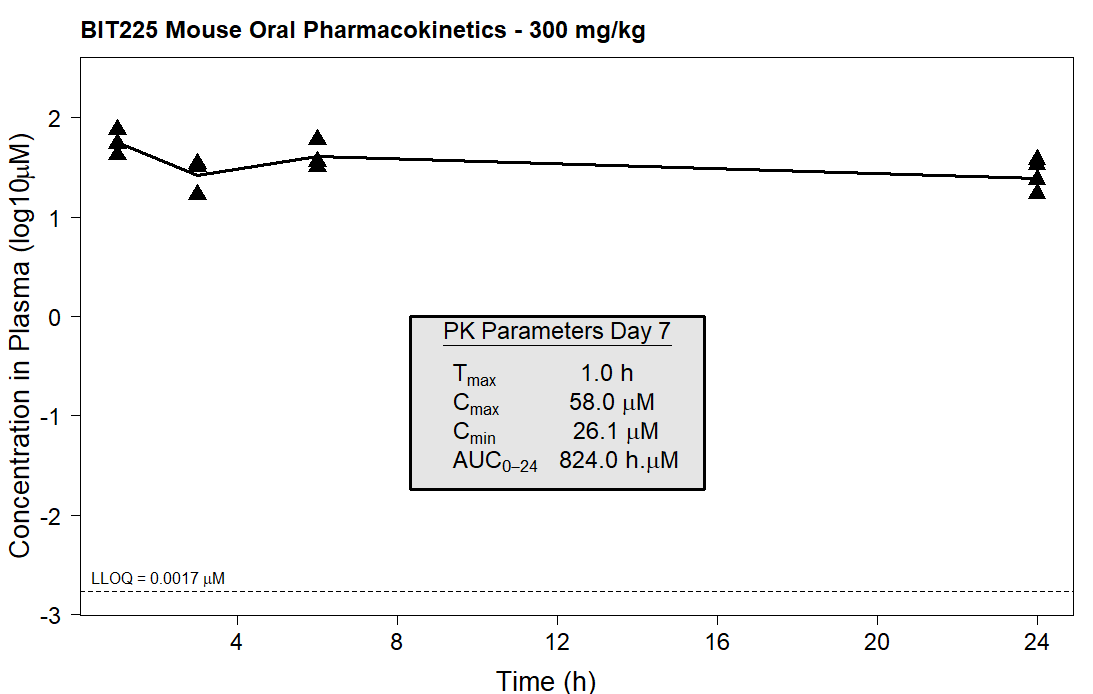

Supplement: S6 Fig — Male outbred Swiss mice (n = 14) were dosed, once daily for 7 days, via oral gavage needle at 3 mL/kg with BIT225 suspended (100 mg/mL) in vehicle (0.5% (w/v) hydroxypropyl methylcellulose, 0.5% (v/v) benzyl alcohol and 0.4% (v/v) Polysorbate 80 in Milli-Q water); giving a total daily dose of 300 mg/kg. On Day 7, blood samples were taken for PK at 1 h, 3 h, 6 h and 24 h, from n = 3, 3, 3 & 5 mice, respectively. The concentration of BIT225 in plasma was measured using a validated LC-MS/MS method. (TIF) [file ppat.1011328.s006.tif]

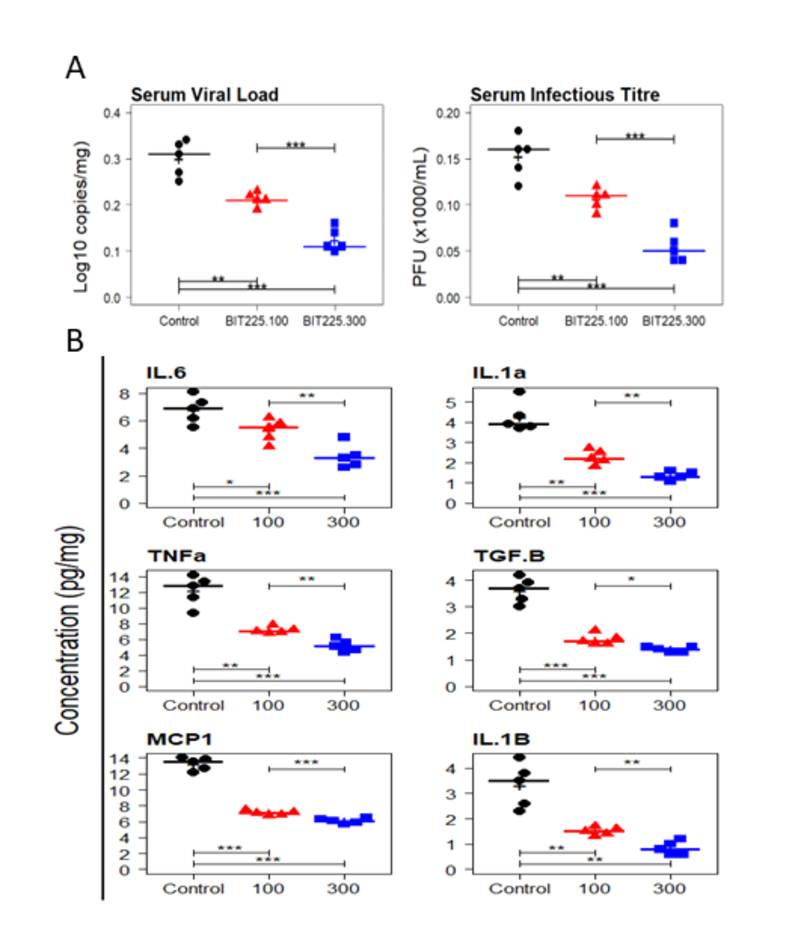

Supplement: S7 Fig — Serum samples were harvested at Day 7 and analysed for; (A) viral load by pRT-PCR assay (left panel) or infectious titre by plaque assay (right panel); and (B) concentration of the indicated cytokines or chemokine by sandwich ELISA assay, as described in Methods. Symbols represent data for individual mice: Vehicle control (black circles); BIT225 (100 mg/kg–red triangles); BIT225 (300 mg/kg–blue squares). Horizontal lines and “+” indicate the group median and mean, respectively. Welch’s T-tests were used to compare the group means and P-values are indicated as: ns—P > 0.05; * P < 0.05; ** P < 0.01; *** P < 0.001. (TIF) [file ppat.1011328.s007.tif]

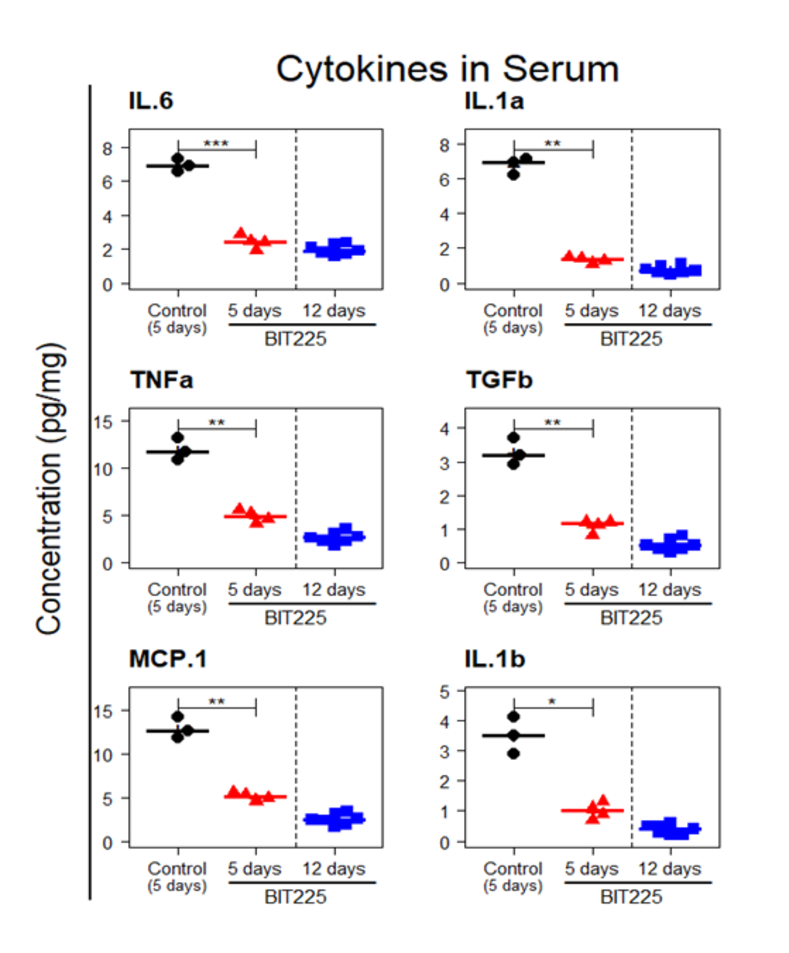

Supplement: S8 Fig — Serum samples were harvested at Day 5 or Day 12 and analysed for concentration of the indicated cytokines or chemokine by sandwich ELISA assay, as described in Methods. Vehicle control and 5-day BIT225 treatment samples were from the satellite group. 12-day data are from the animals that survived to the end of the study. Symbols represent data for individual mice: Vehicle control (black circles); BIT225 5-day group (red triangles); BIT225 12-day group (blue squares). Horizontal lines and “+” indicate the group median and mean, respectively. Welch’s T-tests were used to compare the group means and P-values are indicated as: ns -; P > 0.05; * P < 0.05; ** P < 0.01; *** P < 0.001. (TIF) [file ppat.1011328.s008.tif]

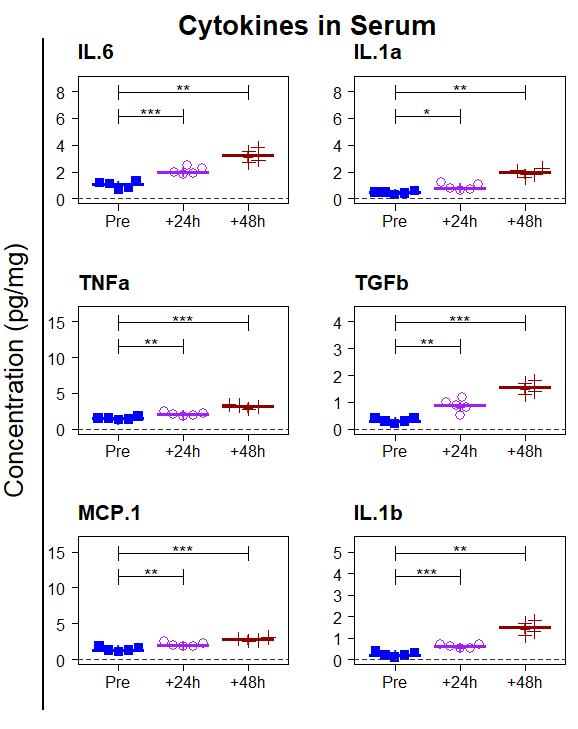

Supplement: S9 Fig — Serum samples were harvested from surviving animals at Day 12 post infection and analysed for concentration of the indicated cytokines or chemokine by sandwich ELISA assay, as described in Methods. No vehicle control animals survived. Symbols represent data for individual mice: Pre-treated group (blue line and squares); 24 h post treated group (purple line and open circles); 48 h post treated group (red line and “+” symbols). Horizontal lines indicate the group median. Welch’s T-tests were used to compare the group means and P-values are indicated as: ns—P > 0.05; * P < 0.05; ** P < 0.01; *** P < 0.001. (TIF) [file ppat.1011328.s009.tif]
