# Supplementary material for: Post-infection treatment with the E protein inhibitor BIT225 reduces disease severity and increases survival of K18-hACE2 transgenic mice infected with a lethal dose of SARS-CoV-2
Source: PLoS Pathog. 2023 Aug 7;19(8):e1011328. doi: 10.1371/journal.ppat.1011328 (PMC10434922; doi:10.1371/journal.ppat.1011328)
Supplement: Attachment — Submitted filename: plos-path-2.pdf [file ppat.1011328.s016.pdf]

The authors have, more or less, satisfactorily addressed the questions of this reviewer on data variations and the large dosages used in the studies described in this manuscript.

A further suggestion for the authors is to discuss the mechanisms of action of BIT225. This is expected to be informative for the following reason: Biotron and collaborators have reported or implicated that the potency and effectiveness of BIT225 against HCV, HIV-1, and SARS CoV-2 were comparable by targeting HCV p7 protein, HIV-1 Vpu, and SARS Cov-2 E protein, respectively. Is BIT225 a privileged binder for diverse targets?
