# Supplementary material for: Post-infection treatment with the E protein inhibitor BIT225 reduces disease severity and increases survival of K18-hACE2 transgenic mice infected with a lethal dose of SARS-CoV-2
Source: PLoS Pathog. 2023 Aug 7;19(8):e1011328. doi: 10.1371/journal.ppat.1011328 (PMC10434922; doi:10.1371/journal.ppat.1011328)
Supplement: Attachment — Submitted filename: EWART et al- Review Response Letter.docx [file ppat.1011328.s015.docx]

56 Delhi Rd, Suite 3.3

North Ryde, Australia, 2113

Mob. +61-(0)429-703301

gewart@biotron.com.au

**Dr Gary Ewart**

Editor(s)

PLOS Pathogens

1/July/2023

Dear Editors,

Following are our responses to the reviewers’ comments/suggestions on our submitted manuscript "Post-infection treatment with the E protein inhibitor BIT225 reduces disease severity and increases survival of k18-hACE2 transgenic mice infected with a lethal dose of SARS-CoV-2."

Below, the reviewers’s comments from Part 1 and Part II - collated by common issue – are reproduced in *italicised text*: Our responses are interspersed in plain text.

**Reviewer #1**:

*The manuscript described an impressive in vivo efficacy of BIT225 against SARS CoV-2 in the K18-hACE2 transgenic mouse model, likely by inhibition of SARS-CoV-2 E-protein viroporin. Although the data are clearly presented and the outcomes of the study are potentially significant, there are some major weaknesses:*

***Summary point #1.*** *The technicality of the animal studies: There were large variations among studies 1-3, including body weight and viral load. For example, the virus caused ~ 5% weight loss in study 1 (1 wk P.I.), but ~ 10% (1 wk P.I.) in other studies. The viral load in lung was close to 2 logs higher in study 1 (day 7) than that of study 2 (day 5). The viral load in lung of SARS CoV-2 infection of K18-hACE2 mice was reportedly to peak as early as day 2 (Nature Immunology, 21: 1327)*

***Major issue #1****. Large variations among studies 1-3: More studies/repetitions are needed to offset the large variations.*

**Response:**

Differences in lung viral load and onset of clinical symptoms after infection are commonly observed when different preparations of virus are used in the K18 mouse model.  We consciously decided to perform three independent in vivo studies for this reason. Importantly, across all three studies the effect of treatment with BIT225 was highly reproducible with respect to protection from weight loss and protection from death as compared to vehicle treated animals. Therefore, we consider it a particular strength of this paper that despite natural differences in virus replication kinetics and kinetics of clinical symptom development, treatment with BIT225 was so robust as to deliver consistently potent inhibition of virus replication and protection from the development of clinical symptoms, severe disease, and death across these independent studies. Each study was independently controlled with vehicle treatment. Within-study variability was typical for this mouse model, and statistical analyses support the findings as presented. More repetitions are not needed to support the conclusions as presented in the manuscript.

***Summary point #2****. Dosage: It is unclear why such a high dose (300 mg/kg BID) was used in most of the studies. BIT225 is roughly equally potent against HIV-1 and SARS CoV-2 in vitro. BIT225 was given to HIV-1 patients at 400 mg/BID in a clinical trial, which is ~50-60-fold lower than that used for the mouse studies described in the manuscript. A PK study from some of the authors indicated that the clinical trial dosage resulted in a steady state plasma concentration > 5 EC50. BIT225 was also tested for efficacy in an HIV-1 mouse model, where much lower dose (1/6 of this study) was used to show efficacy by some of the authors.*

***Major issue #2****. High dosage: Justification by either PK data or show a dose-dependent efficacy study with multiple dosages.*

**Response:**

The two doses (100 mg/kg and 300 mg/kg) used in these studies were chosen based on prior PK experiments in Swiss outbred mice. Based on allometric scaling the 100mg/kg BID dose in mouse corresponds to ~1.4-fold that of a 400 mg BID dose in human and was therefore taken as a starting dose in the PK studies, together with a 3-fold higher dose. The results from the PK assessment in mice dosed with the 300mg/kg dose showed a mean Cmax (n=3) of 46.2 µM on Day 1, and 58 µM after 7 days of repeated daily dosing. The plasma concentration at 24 hours (Cmin) was 26.1 µM on Day 7. In humans dosed at 200 mg BID with BIT225, steady state Cmax and Cmin were 20 µM and 13 µM, respectively.  These data suggest that a 300mg/kg dose in mice delivers plasma exposures similar to those that could be achieved in human with BIT225 doses that have demonstrated safety and efficacy in clinical trials.  Furthermore, and as noted in the manuscript, a clinical study of BIT225 treatment of SARS-CoV-2 infection will soon commence and future development of BIT225 in SARS-CoV-2 will be guided by results from this trial.

Also, as already presented in this manuscript, the comparison of 100 mg/kg and 300 mg/kg once-daily dosing in the K18 mouse model, showed significant protection from clinical symptoms in both dose groups, with a superior performance of the 300 mg/kg dose group. No adverse effects were observed with either dose level. Therefore, based on the superior efficacy in the mouse model and on the PK data in mice that showed clinically achievable and safe exposures in the mouse, the 300mg/kg dose was used in this model going forward to Studies 2 and 3.

Supplementary Fig 6 has been added to present the mouse PK data. Also, the following text has been added to the results section:

Line 181: words ‘and dose response’ added to the title: BIT225 efficacy and dose response when treatment is initiated before infection.

Lines 184-190: text added: The two dose levels were selected based on mouse pharmacokinetics data. The high dose of 300 mg/kg gave mean plasma BIT225 C_max_ and C_24h_ concentrations of 58 µM and 26 µM, respectively on day 7 (Supplementary Fig 6). These plasma concentrations were above concentrations required to inhibit SARS-CoV-2 replication in vitro and similar to plasma concentrations of BIT225 that have been achieved in clinical trials in human. A lower dose was also included in the mouse model to assess dose dependence of antiviral activity *in vivo*.

The following text has been inserted in the discussion section (line 359-366):

“The dose of 300 mg/kg used in the current mouse study was well tolerated in both outbred Swiss mice (used in the preliminary PK studies) and infected K18-hACE2 mice; in the latter case providing not only survival from SARS-2 infection, but also supporting normal weight gain over 12 days of daily dosing. This dose of oral BIT225 in mice delivered a mean C_max_ of 58 µM and C_24h_ of 26 µM after 7 days of once daily dosing (Supplementary Fig 6). This is comparable to plasma concentrations observed in human clinical trials where steady state C_max_ and C_min_ of 20 µM and 13 µM, respectively were achieved with 200 mg BID dosing.”

Figure 2 and Supplementary Fig 7 show the dose-dependent efficacy of BIT225 in the K18 mouse model. To highlight this, we have inserted the words “dose-dependence” into the titles of figure legends for these figures.

The following text has been added to the discussion section (lines 275-282): Treatment efficacy of BIT225 was dependent on dose (see Fig 2 and Supplementary Fig 7): In study 1, viral loads, infectious titres and inflammatory cytokine levels were significantly lower in lung and serum samples from the 300 mg/kg group than the 100 mg/kg group. The dose-dependent antiviral effect was consistent with a higher margin of plasma exposure relative to intrinsic antiviral potency of BIT225 in the 300mg/kg dose group. Both dose groups effectively blocked virus replication and prevented clinical signs such as body weight loss as compared to vehicle control. Based on the more robust antiviral efficacy in the mice, the 300 mg/kg dose was used going forward to the follow-up studies 2 and 3.

**Reviewer #2**:

***Summary:*** *The authors of this work describe the potential use of the clinical stage viroporin inhibitor BIT225 as a SARS-CoV-2 therapeutic. SARS-CoV-2 has four viroporin-like proteins that it encodes (Envelope (E), Orf 3a, Orf 7b, and Orf 10). The authors demonstrate that BIT225 impairs the viroporin activity of E, and that low µM concentrations of the drug are capable of inhibiting infection by multiple SARS-CoV-2 variants in two cell culture systems (Vero and Calu3). These values are similar to those attained against HIV, but about 10-30x higher than those for BVDV/HCV. The authors use the K18-hACE2 mouse model to show that BIT225 reduces in vivo infection and inflammation. BIT225 is able to reduce morbidity, mortality, and inflammation associated with SARS-CoV-2 in vivo when given either prophylactically or therapeutically. The paper is well-written and the implications of conclusions are not overstated. While BIT225 is itself not novel, there is innovation in the re-application to SARS-CoV-2 and potential promise of an alternative therapeutic strategy. However, the manuscript could be improved with the addition of some controls and experimentation.*

***Major issue #1a****. Please include the other SARS-CoV-2 viroporins in the Xenopus oocyte experiment represented in Figure 1.*

**Response:**

We agree with the reviewer that it would be highly interesting to determine ion channel activities and the impact of BIT225 on these for the other proposed viroporins of SARS-CoV-2.  However, current techniques make such experiments challenging; reliable methods to measure these activities still have to be established. We believe such studies go beyond the scope of this manuscript, as it is not the intention of this manuscript to determine the detailed mechanism of action of BIT225 here. While we see that BIT225 is an inhibitor of E-protein ion channel activity, we specifically mention in this manuscript that the particularly high in vivo efficacy of BIT225 may stem from simultaneous inhibition of multiple viroporins. Indeed, it will be interesting to find out more about this point in the future.

***Major issue #1b****. Please also include non-viroporin/ion channel controls to ensure that injection of the oocyte itself/the translation of incoming mRNA doesn't lead to significant changes in resting membrane potential.*

**Response:** As per the reviewer’s request, we have determined and compared the resting membrane potential between un-injected, water injected (50 nL) and cRNA injected (20 ng in 50 nL) oocytes recorded by two-electrode voltage-clamp following 3 days of incubation. As a non-viroporin control, we used the cRNA encoding the non-pore-forming protein potassium channel interacting protein 2, the cytoplasmic auxiliary subunit, KChIP2.1. The resting membrane potential was not different between un-injected, water injected and KChIP2.1 cRNA injected oocytes. This data has been added as a supplementary Figure 5.

The following wording has been added to the results section, lines 168-170:

‘Oocytes injected with cRNA encoding a non-pore-forming control protein, the potassium channel interacting protein 2, KChIP2, showed resting potential similar to uninjected and water-injected controls (Supplementary Fig. 5).’

***Major issue #2:*** *There is little data that suggests the only target of BIT225 against SARS-CoV-2 is the E viroporin. Please perform passaging experiments of SARS-CoV-2 to generate escape mutants and genetically map the escape mutations to present stronger evidence that it is Envelope that BIT225 antagonizes.*

**Response:**

We agree that virus passaging is of high scientific interest, and should the clinical trial demonstrate efficacy, will be included for later-stage phase II and phase III studies, but this goes beyond the scope of this manuscript. As mentioned above, such studies will be done, because other viroporins may also be inhibited by BIT225. Such studies are extensive and require substantial resources and time. We aim to pursue such studies and will publish results separately in due course. As mentioned in the manuscript, we do NOT suggest that E is the only target of BIT225, and we do suggest that the very robust in vivo efficacy of BIT225 treatment may indeed be associated with a synergy in vivo derived from inhibition of multiple viroporins by BIT225.

***Major issue #3a:*** *For the in vivo data in Figure 2, please include no-BIT225/no-SARS-CoV-2 and BIT225 treatment alone controls to establish the impact of BIT225 on morbidity/mortality and inflammation.*

The reviewer suggests evaluating ‘no BIT225/no SARS-CoV-2 mice', i.e., uninfected, untreated mice, i.e., healthy mice, for morbidity / mortality and inflammation. Healthy mice are commonly handled in the in vivo laboratory and generally do not show the consistent weight loss and high levels of inflammation markers observed here.  We suggest that a healthy, uninfected, untreated mouse group is not required to support the conclusions as described in this manuscript.

The reviewer suggests including a 'BIT225 treatment alone group', i.e., uninfected, treated mice, for morbidity / mortality and inflammation assessment.  Such a group would indeed be important if BIT225 treatment itself may be associated with toxicity such that morbidity / mortality could be derived from treatment rather than infection.  Also, certain treatments may be immune activating such that inflammation markers may be elevated by treatment itself rather than infection.  We suggest that a BIT225 treatment alone group in uninfected mice is not required in this case, because the treatment of infected mice strongly PREVENTED morbidity and mortality and REDUCED inflammation markers. The clinical observations show that BIT225 treatment was exceptionally well tolerated, such that there is no ambiguity about the virus infection as the cause of morbidity and mortality and increased inflammation markers observed in this study. Therefore, the inclusion of a BIT treatment alone group would not change the conclusions as presented in this manuscript and is not required in this study.

***Major issue #3b:*** *The authors should include experiments combining drug regimens (as they mention for other SARS-CoV-2 antivirals (lines 307-308) as well as BIT225 (lines 334-336)). Previous work in BVDV showed significant synergism of BIT225 with the multiple anti-HCV/BVDV interventions; as BIT225 has a much greater EC50 against SARS-CoV-2 than BVDV, synergism that allows for greatly reduced levels of BIT225 would be desired.*

**Response:**

The results shown here demonstrate the ability of BIT225 MONOTHERAPY to completely prevent weight loss and mortality in the K18 mouse model, under conditions where all untreated mice died within 5-9 days after infection. Antiviral combinations could not further improve clinical outcomes.   For future studies it would be of interest to evaluate drug combinations with BIT225 to evaluate if combinations could reduce the dose / exposure requirements for BIT225, or if combinations could allow later start of treatment after infection. Combination studies will be considered in the future following the outcome of the pending clinical study of BIT225 monotherapy. We propose such studies are beyond the scope of this manuscript.

**Part III – Minor Issues: Editorial and Data Presentation Modifications**

**Reviewer #1:** (None)

**Reviewer #2:** *Line 337-338* (original numbering)*- the authors state "BIT225 showed similar antiviral activity against six different SARS-CoV-2 strains, supporting the broad-spectrum potential across coronaviruses." As the authors only test SARS-CoV-2 and no other coronaviruses, this sentence should be re-stated "...supporting the broad-spectrum potential against SARS-CoV-2 variants."*

**Response:**

We changed this sentence as proposed by the Reviewer (line 354 in revised manuscript).

---------

Finally, as required, we have uploaded two versions of the revised manuscript:

- EWART et al- BIT225 v hACE2 Mouse_Rev3_clean.docx: This is the new manuscript file.
- EWART et al- BIT225 v hACE2 Mouse_Rev3_marked.docx: This shows modifications to the text highlighted in blue or red coloured font.

Figures have been processed through the ‘PACE’ site and uploaded. An Excel file, containing raw data values, has also been uploaded.

We hope you agree that all reviewers’ comments have been appropriately addressed and look forward to your decision on the revised manuscript.

Sincerely,

Gary Ewart

Dr Gary Ewart - Senior Scientist – Biotron Limited
